# Decompressive Craniectomy in Pediatric Patients

**DOI:** 10.3389/fsurg.2022.860896

**Published:** 2023-06-14

**Authors:** Soham Bandyopadhyay, Ryan Gidda, Noel Peter, Kokila Lakhoo

**Affiliations:** Oxford University Global Surgery Group, Medical Sciences Division, Nuffield Department of Surgical Sciences, University of Oxford, Oxford, United Kingdom

**Keywords:** pediatric, traumatic brain injury, decompressive craniectomy, evidence based medicine, neurosurgery

Childhood injuries are the leading cause of death in children above the age of one (1, 2). Among all the injury types, traumatic brain injury (TBI)—defined as “an alteration in brain function or other evidence of brain pathology, caused by an external force” (3)—is the most likely to result in death or permanent disability of a child (2). In addition, TBI contributes to global mortality and morbidity more than any other traumatic insult (4). The inordinately high number of person-years of life lost make pediatric TBI (pTBI) a public health priority (5). Despite this high prevalence of pTBI, there is limited evidence on which to base protocols for management. Most of the current treatment algorithms are founded on research in adults (6).

Current Brain Trauma Foundation guidelines suggest that decompressive craniectomy (DC) is a potentially suitable therapeutic measure for certain patients with TBI (6). The evidence base to support this is largely from trials in adults (7, 8), with limited evidence for this surgical procedure in children. The earliest study into the utility of DC for pTBI was by Polin et al. (9). Their retrospective cohort study found that pTBI patients who underwent DC were significantly more likely to have favorable outcomes (moderate disability or better) than patients who underwent medical management alone. However, this study's application to current practice is limited by its small sample size (*n* = 18), lack of control for confounding factors that may have resulted in different treatment modalities being utilized, and medical management that is not representative of contemporary pTBI management. Unfortunately, there have been few studies since then on this topic. Those that have been conducted have largely been limited due to their retrospective nature and small sample size (10, 11). Only one randomized trial (RCT) has been conducted in this field (12). In 2001, Taylor et al. randomly assigned 27 patients to craniectomy or medical management alone. They found that the craniectomy patients obtained better outcomes (54 vs. 14%). However, statistical significance was not met because of the small sample size. The quality of the evidence was also decreased by biases introduced from the trial terminating early, the allocation method changing midway through which failed to conceal participant allocation, the surgical procedure leaving the dura mater intact, and the short follow-up time.

High-quality multi-center RCTs focusing on pTBI are urgently needed. France is paving the way in this matter, having recently launched the “Decompressive Craniectomy for Severe Traumatic Brain Injury in Children With Refractory Intracranial Hypertension” (RANDECPED) trial (13). If the methodology outlined in the protocol is followed and a sufficient sample size are recruited, it should add valuable evidence. However, the transferability of results may be limited due to differences between settings. Therefore, centers in other countries should also look to carry out their own controlled studies. Until further high-quality evidence is obtained, clinicians should ensure that parents are sensitively given all the information necessary to understand the element of uncertainty of the procedure and the long-term changes in functional outcome before operating. Understanding the clinical significance of DC as a procedure involves appreciating the priorities of both participants and their families and incorporating this into how we judge it as an effective treatment.

## Author Contributions

All authors listed have made a substantial, direct, and intellectual contribution to the work and approved it for publication.

## Funding

SB is funded by the National Institute for Health Research (Academic Clinical Fellowship 2022-26-004).

## Conflict of Interest

The authors declare that the research was conducted in the absence of any commercial or financial relationships that could be construed as a potential conflict of interest.

## Publisher's Note

All claims expressed in this article are solely those of the authors and do not necessarily represent those of their affiliated organizations, or those of the publisher, the editors and the reviewers. Any product that may be evaluated in this article, or claim that may be made by its manufacturer, is not guaranteed or endorsed by the publisher.
